# Supplementary material for: Association between oral microbiome and seven types of cancers in East Asian population: a two-sample Mendelian randomization analysis
Source: Front Mol Biosci. 2023 Nov 21;10:1327893. doi: 10.3389/fmolb.2023.1327893 (PMC10702768; doi:10.3389/fmolb.2023.1327893)
Supplement: Supplementary file 1 [file DataSheet1.ZIP › Supplementary Materials/MR plots of saliva/saliva═╝/Pancreatic cancer/pheno.2084_to_pancreatic cancer_leave_one_out.pdf]

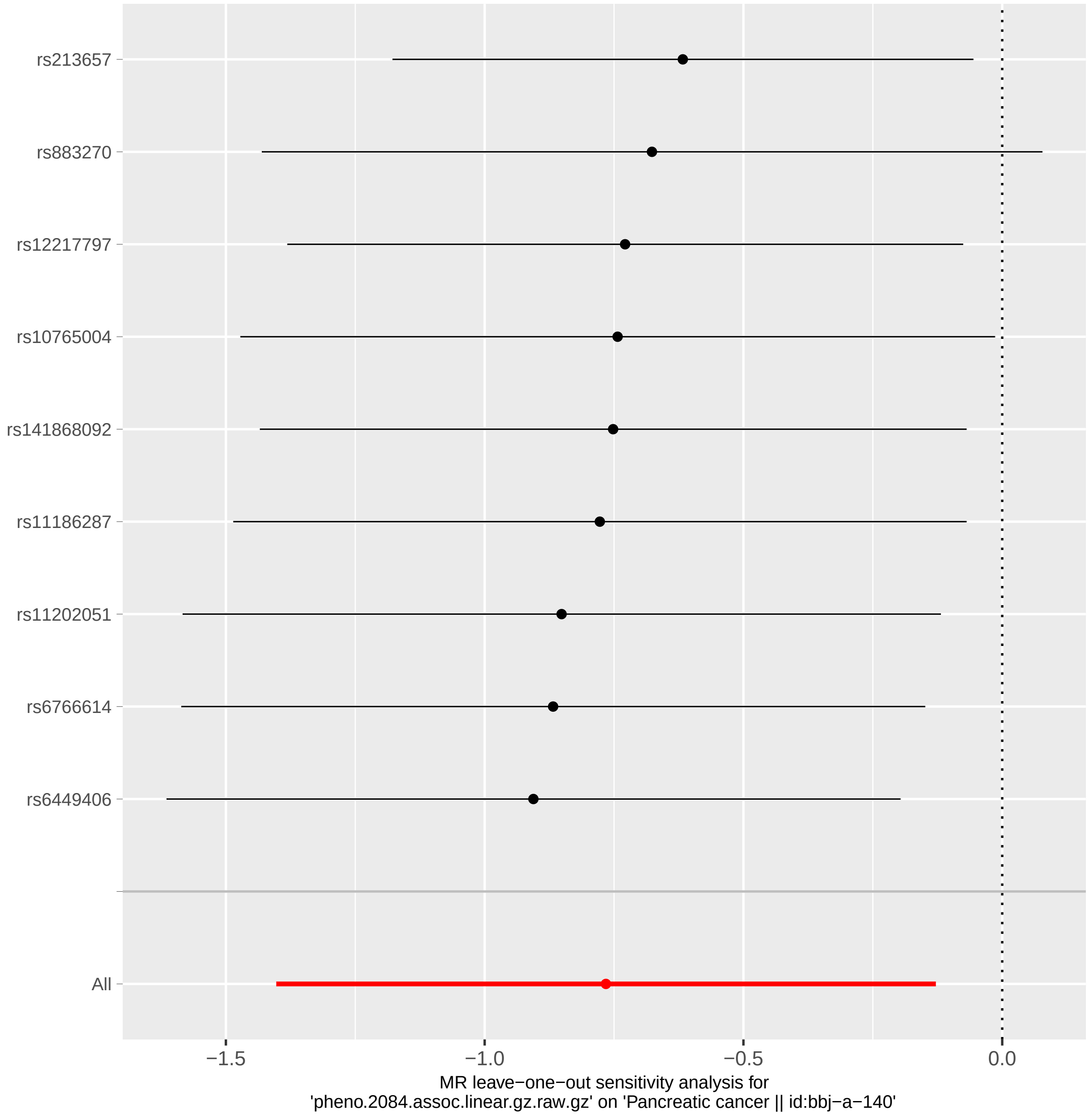
rs213657
rs883270
rs12217797
rs10765004
rs141868092
rs11186287
rs11202051
rs6766614
rs6449406
All
−1.5
−1.0
−0.5
0.0
MR leave−one−out sensitivity analysis for
'pheno.2084.assoc.linear.gz.raw.gz' on 'Pancreatic cancer || id:bbj−a−140'
